# Training in psychiatry and gender equality: how to improve it?

**DOI:** 10.1192/j.eurpsy.2026.10274

**Published:** 2026-07-31

**Authors:** M. Casanova Dias

**Affiliations:** 1 Southwark Perinatal Community Team, South London and Maudsley NHS FT; 2 King’s Women’s Mental Health, King’s College London, London, United Kingdom; 3 Section of Psychiatry, European Union of Medical Specialists (UEMS), Brussels, Belgium

## Abstract

**Abstract:**

Psychiatry sees itself as progressive, yet gender inequality persists within our training systems — subtle, structural, and rarely challenged. Women enter the field in equal or greater numbers, but senior leadership remains disproportionately male. This is not just a pipeline problem; it is a training problem.

Training transmits not only knowledge but norms. What we teach — and omit — shapes who advance and which work is sidelined.

First, the content of training itself often renders gender peripheral. Sex- and gender-sensitive psychiatry is frequently confined to optional modules or niche subspecialties such as perinatal mental health. Core curricula still prioritise “neutral” diagnostic frameworks that implicitly centre male presentations of illness. Gender-based violence, reproductive transitions, caregiving burdens, and intersectional discrimination are treated as contextual add-ons rather than central determinants of mental health. When gender is framed as a “special interest,” those who champion it risk being labelled advocates rather than scientists — a subtle but powerful professional devaluation.

Second, training reinforces unexamined professional norms. Trainees quickly learn which research topics are considered prestigious and career-enhancing or invisible labour. Teaching, mentoring, pastoral support, and diversity work — disproportionately undertaken by women — are rarely rewarded in promotion criteria. Meanwhile, assertiveness in leadership is praised in men and problematised in women. These double standards are not written into curricula, but they are learned experientially.

Third, academic training structures often clash with reproductive timelines. Fellowship schemes and publication metrics assume uninterrupted productivity during years when many trainees take on parental or caring responsibilities. Flexibility is framed as accommodation rather than entitlement, reinforcing the idea that caregiving deviates from the professional norm — and in some countries, is barely supported at all.

If psychiatry is serious about gender equality, training reform must be structural, not symbolic. This means embedding sex- and gender-based science across curricula, integrating intersectionality into core teaching, valuing educational and equity work in assessments, ensuring transparent authorship and sponsorship, and publishing gender-disaggregated data on research, training, and promotion outcomes.

Early-career psychiatrists are uniquely positioned to challenge entrenched norms because they experience them most acutely. But change cannot depend on individual resilience; it requires institutional accountability. Gender equality will not arise from demographic shifts alone. Without redesigning training, evaluation, and culture, we will reproduce the hierarchies we critique. Reforming psychiatric training is not a diversity add-on — it is a scientific, ethical, and professional necessity.

**Disclosure of Interest:**

None Declared

